# Dissipation of oscillatory contact lines using resonant mode scanning

**DOI:** 10.1038/s41526-019-0093-0

**Published:** 2020-01-21

**Authors:** Yi Xia, Paul H. Steen

**Affiliations:** 1 Sibley School of Mechanical and Aerospace Engineering, Cornell University, Ithaca, NY 14853 USA; 2 Robert Frederick Smith School of Chemical and Biomolecular Engineering, Cornell University, Ithaca, NY 14853 USA

**Keywords:** Thermodynamics, Fluid dynamics

## Abstract

Moving contact-lines (CLs) dissipate. Sessile droplets, mechanically driven into resonance by plane-normal forcing of the contacting substrate, can exhibit oscillatory CL motions with CL losses dominating bulk dissipation. Conventional practice measures CL dissipation based on the rate of mechanical work of the unbalanced Young’s force at the CL. Typical approaches require measurements local to the CL and assumptions about the “equilibrium” contact angle (CA). This paper demonstrates how to use scanning of forcing frequency to characterize CL dissipation without any dependence on measurements from the vicinity of the CL. The results are of immediate relevance to an International Space Station (ISS) experiment and of longer-term relevance to Earth-based wettability applications. Experiments reported here use various concentrations of a water-glycerol mixture on a low-hysteresis non-wetting substrate.

## Introduction

The contact line (CL) is the idealized boundary between wet and unwet support. The molecular-scale origin of macro-scale behavior of moving CLs remains a subject of debate (see ref. ^1^ for a recent review). Dissipation owing to CL motion is ultimately of molecular-scale origin yet remains challenging to measure, especially as distinguished from other dissipation contributions. Here we introduce a simple measurement approach.

To what extent can macroscopic measurements away from the CL characterize the dissipative behavior arising from inherently fast and small scale events near the moving CL? We introduce the CL damping ratio and show that it provides a practical guide to wettability characterization.^2^ Using this metric, we also illustrate how overall damping partitions between the CL and bulk dissipation mechanisms for an exemplary system.

Applications where CL dissipation can play an important role include self-cleaning surfaces,^3^ anti-frost coatings^4^ and water harvesting devices,^5^ which typically involve dynamical droplet phenomena on hydrophobic and superhydrophobic surfaces. For dropwise condensation heat transfer devices, the transition between dropwise and filmwise heat transfer is crucial and strongly influenced by moving CLs.^6,7^ Finally, to the extent that applications involving spray painting of large areas depend on droplet impacts, spreading, and droplet mergers for a uniform coat, CL dissipation can also be important.

Various authors have examined energy dissipation arising from CL motion. In the context of rivulets, ref. ^8^ shows via linearized stability theory that dissipation can arise solely from CL motion, as distinguished from bulk viscous dissipation. Reference ^9^ proposed the form of the local dissipation per unit length at the CL,

1 $${\mathcal{D}}={\oint }_{{\!\!\!\rm{CL}}}{\mu }_{{\rm{f}}}{U}_{{\rm{CL}}}^{2}ds,$$

where ${\mu }_{{\rm{f}}}$ is a friction coefficient and ${U}_{{\rm{CL}}}$ is the rms CL velocity and the integration is with respect to arclength around the closed CL. In the context of an oscillating drop, ^10^ use matched asymptotics as well as a pseudo-spectral method to illustrate theoretically that CL dissipation is important and sometimes dominates over viscous dissipation. More recently, numerous authors^11–13^ have adopted the form of Eq. (1), which is reminiscent of the local viscous dissipation due to viscosity $\mu$. Note that ${\mu }_{{\rm{f}}}$ and $\mu$ have the same dimensions.

Experimentally, CL dissipation remains difficult to measure. Most do so by measuring the mechanical work done by the unbalanced Young’s force per unit length along the CL,

2 $$F=-\sigma \left\{\cos \alpha -\cos \overline{\alpha }\right\},$$

where $\sigma$ is the liquid-gas surface tension and $\overline{\alpha }$ is the mean CA.^9^ Note that $\overline{\alpha }$ need not coincide with the “equilibrium” angle. The dissipation per unit length of CL is evaluated equating the dissipation defined by Eq. (1) to the rate of mechanical work done by $F$,

3 $${\mathcal{P}}={\oint}_{{\!\!\!\rm{CL}}}F\ {U}_{{\rm{CL}}}ds,$$

or, equivalently, the power exerted at the CL. Here, the integration is around the closed CL.

Reference^13^ evaluates ${\mathcal{P}}$ using images from short-time dynamic wetting experiments. Eschewing direct optical measurements of the CA, ref. ^14^ opt to measure the capillary pressure drop in a cylindrical tube as a proxy for evaluating the CA. Reference^15^ use a custom-built tensiometer to measure $F$ directly, circumventing the need for CA measurement altogether but still requiring some assumption about the equilibrium CA to calculate the microscopic friction force. By and large, considerations of CL dissipation based on mechanical work require measurement of the CA as well as some assumption about its “equilibrium” value. In itself, the latter may be problematic because stick-slip can leave the CL hung up in various positions yielding various associated CAs.^16–18^ Identifying *the* equilibrium CA is not needed in our approach.

All experiments reported above are Earth-based, wherein capillary length-scales (CLS), $\sqrt{\sigma /\rho g}$, are on order of a few millimeters, at largest. The CLS practically defines what is meant by “vicinity of the CL” since, at larger scales, shape deformations include the influence of gravity and thus complicate direct measurement of a pure Young’s force. That is, above the CLS, gravity can contaminate direct measurement of Eq. (2).

Corresponding to the low-gravity of the ISS, there is a large CLS. This has a primary benefit of allowing greater resolution for the same measurement device by using larger liquid drops. A secondary benefit is that the time-scale of inertial-capillary oscillations is amplified by $\sqrt{V}$, effectively shifting natural frequencies lower. The ISV experiments, to be summarized later, take advantage of larger, slower drops to make measurements of CL and CA motions for evaluation of CL dissipation according to the methods introduced in ref. ^19^ and demonstrated in this paper. Our apparatus is presently scheduled to be conveyed to the ISS in 2020 for subsequent experiments.

Our purpose below is to demonstrate that the highly resolved measurements needed to evaluate (3) directly can be circumvented. Our circumvention will rely on driving sessile drops near resonance and observing the cyclic drop apex motion. The motions are underdamped—inertia and capillarity dominate bulk viscosity. To substantiate our circumvention claim, we will also compare to direct near CL measurements.

**Fig. 1:**
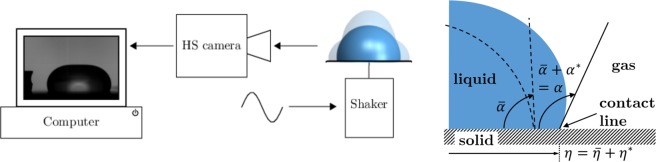
**a** Experimental setup. **b** Definition sketch: instantaneous configuration (colored) and time-mean configuration (dashed), with instantaneous, mean and deviation CA and CL displacement, related as $\alpha =\overline{\alpha }+{\alpha }^{* }$ and $\eta =\overline{\eta }+{\eta }^{* }$, respectively.

The Earth-based experimental setup, Fig. 1a, includes a droplet shaker to excite oscillatory drop motion, a high-speed camera for image capture, and a computer for subsequent edge detection and analysis. Relevant dimensionless numbers are given in Table 1 for the system under investigation. Inertial-capillary flow, ${{\rm{Re}}}_{{\rm{f}}}\ >\ 1$ and ${{\rm{Ca}}}_{{\rm{o}}}\ll 1$, characterizes the range of our experiments where “${\rm{f}}$” and “${\rm{o}}$” distinguish between “forced” and “observed at the CL” and $a$ is the forcing acceleration.

**Table 1:** Dimensionless numbers for system W00 (Table 4).

Symbol	${\rm{Oh}}$	${{\rm{Re}}}_{{\rm{f}}}$	${{\rm{Bo}}}_{{\rm{f}}}$	${{\rm{Re}}}_{{\rm{o}}}$	${{\rm{Ca}}}_{{\rm{o}}}$	${{\rm{We}}}_{{\rm{o}}}$
Definition	$\frac{\mu }{\sqrt{\rho D\sigma }}$	$\frac{a\rho D}{\mu \omega }$	$\frac{a\rho {D}^{2}}{\sigma }$	$\frac{\rho \overline{{U}_{{\rm{CL}}}}D}{\mu }$	$\frac{\mu \overline{{U}_{{\rm{CL}}}}}{\sigma }$	$\frac{\rho {\overline{{U}_{{\rm{CL}}}}}^{2}D}{\sigma }$
Values	(2.1–2.5)e-3	1.3–43	0.017–0.60	0.67–53	(3–240)e-6	2.0e-6–0.012

subscript f = “forcing”, subscript o = “observed”, volume-length $D\equiv {V}^{1/3}$, $\overline{{U}_{{\rm{CL}}}}= d\eta/dt$, Fig. 1b, and ref. ^19^

Interpretation of the response will depend on a reduced-order mechanical model consisting of the mass-spring-damper. We will test the extent to which CL dissipation can be distinguished from bulk dissipation, whether from the viscous layer adjacent to the contact plane or from viscous behavior otherwise in the bulk droplet. In systems where CL dissipation dominates, the technique demonstrated may be used to estimate CL dissipation while, more generally, it may be used to complement other techniques by corroborating those measurements. In both cases, simplicity and statistics over many cycles are major advantages.

We begin by defining per-cycle operations that aid interpretation of the cyclic time-series responses and follow with background on the mass-spring-damper model. Next, we present and discuss our main results and offer some discussion. Finally, details of the methods are provided.

## Results

### Cyclic axially-symmetric response

Measurement over many cycles is important to characterizing oscillatory CL behavior. To that end, we define the time-mean of a quantity $h(t)$ over the cyclic period $T$ and related quantities,

$$\begin{array}{l}\overline{h}\equiv \frac{1}{T}\displaystyle{\int_{nT}^{(n+1)T}}h(t)dt,\\ | h| \equiv \sqrt{2}\ \ {(\overline{{h}^{2}})}^{1/2},\ {h}^{* }\equiv h-\overline{h},\\ \Delta h\equiv \mathop{{\rm{max}}}\limits_{nT\le t < (n+1)T}\ {h}^{* }-\mathop{{\rm{min}}}\limits_{nT\le t<(n+1)T}\ {h}^{* }\end{array}$$

where $n$ is the index of the cycle. Consider the CA, for instance, among relevant variables defined in Fig. 1b. It can be split into its time-mean $\overline{\alpha }$ and deviation from the mean, ${\alpha }^{* }$, $\alpha \equiv \overline{\alpha }+{\alpha }^{* }$. We will also need the maximum of CA deviation over a cycle, $\Delta \alpha$, and similar quantities for the CL displacement.

For a drop in cyclic axisymmetric motion with footprint radius $r(t)=\eta$, and Eq. (3) simplifies to

4 $${\mathcal{P}}=2\pi rF\dot{\eta },$$

using $\dot{\eta }\equiv {U}_{{\rm{CL}}}$. Viewing $F$ as the physical driving force for CL motion, the directions of $F$ and $\dot{\eta }$ must agree and hence the sign of Eq. (4) is non-negative. The time mean of Eq. (4) is

5 $$\overline{{\mathcal{P}}}=2\pi\, \overline{rF\dot{\eta }}.$$

Also for a cyclic axisymmetric response, Eq. (1) similarly becomes

6 $$\overline{{\mathcal{D}}}=2\pi {\mu }_{{\rm{f}}}\,\overline{r{\dot{\eta }}^{2}}.$$

An important interpretation of these time-mean operations is that, for any integrand of the form $g\dot{\eta }$, the integral over any period is the area ${\mathcal{A}}$ of the region ${D}_{\eta g}$ enclosed by a curve ${\mathcal{C}}$ in the $\eta g$-plane,

7 $$T\, \overline{g\dot{\eta }}={\oint_{{\mathcal{C}}}}g\, d\eta ={\iint_{\!{D}_{\eta g}}}\ d\eta\, dg\equiv {\mathcal{A}}[{D}_{\eta g}],$$

where the second equality follows by Green’s theorem. By choosing $g\;=\;rF$ and, alternatively, $g=r\dot{\eta }$ in Eq. (7), the following are immediate, respectively,

8 $$\overline{{\mathcal{P}}}\, T=2\pi\, {\mathcal{A}}[{D}_{\eta rF}],\quad\overline{{\mathcal{D}}}\, T={\mu }_{{\rm{f}}}\,2\pi\,{\mathcal{A}}[{D}_{\eta r\dot{\eta }}].$$

Equation (8) gives the per-cycle mechanical work done and energy dissipated. Plots of data curves ${\mathcal{C}}$ in the $\eta rF$- and $\eta r\dot{\eta }$-planes with enclosed ${\mathcal{A}}$ are used below to estimate ${\mathcal{P}}$ and ${\mathcal{D}}$ at the CL. In the small CL deviation limit, $| {\eta }^{* }| \ll \overline{\eta }$, which for circular CLs is equivalently written $| {r}^{* }| \ll \overline{r}$ obtained by putting $r=\overline{r}+{r}^{* }$ in Eq. (8) and neglecting higher-order effects while writing $R\equiv \overline{r}$ for convenience in notation,

9 $$\overline{{\mathcal{P}}}\approx {\mathcal{A}}[{D}_{\eta F}]\ \ 2\pi R/T,\,\,\,\,\overline{{\mathcal{D}}}\approx {\mathcal{A}}[{D}_{\eta \dot{\eta }}]\ \ {\mu }_{{\rm{f}}}\ \ 2\pi R/T.$$

Below, we also choose a material system that conveniently accommodates the approximation of ${F}^{* }\approx \sigma \sin \overline{\alpha }\ \ {\alpha }^{* }$ in $F=\overline{F}+{F}^{* }$ to further obtain,

10 $$\overline{{\mathcal{P}}}\approx {\mathcal{A}}[{D}_{{\eta }^{* }{\alpha }^{* }}]\ \ 2\pi R\sigma \sin \overline{\alpha }/T.$$

The ${\eta }^{* }{\alpha }^{* }$ diagram is reproduced below in Fig. 5a and related plots are discussed in ref. ^19^ To the authors’ knowledge, the derivation of Eq. (7) and the resulting relations Eq. (8) are new.

### Interpreting frequency scans

**Fig. 2:**
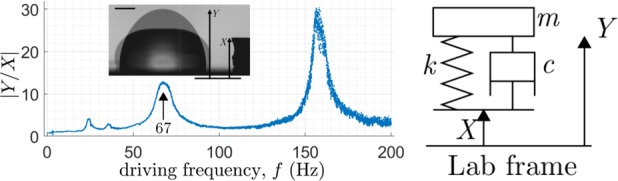
**a** Frequency scan of $20\ \mu$L water drop, with peaks typical of underdamping. Inset: shape extremes, scale bar equals 1 mm. **b** Damped harmonic oscillator: mass $m$ of displacement $Y$ attached via spring $k$ and damper $c$ to moving platform of displacement $X$.

The variation of the normalized apex displacement $| Y/X|$ is plotted against frequency $f$ in Fig. 2a. At resonance, an undamped oscillator would have an infinite peak whereas as damping increases the peak would lower and broaden until below critical damping where peaks would disappear. Our water drops respond as underdamped oscillators.

The 67 Hz peak has a corresponding shape with one nodal circle, as evident in the image, while the shape at 155 Hz shows two nodal circles. These correspond to two- and three-layer bouncing modes, respectively, or $[2,0]$ and $[4,0]$ modes in the nomenclature of,^20^ two of some 35 sessile drop modes discovered to date.^21^ The relative amplitude of the two prominent peaks may be surprising—the lower (67 Hz) is much lower in amplitude than the higher peak (155 Hz), suggesting greater damping, which^19^ attribute to greater CL dissipation owing to greater CL motion. Less prominent peaks (23 Hz, 35 Hz) are subharmonic resonances.

Our interpretation of the droplet response builds upon a classic theorem of mechanics, which states that “the motion of the center of mass (CM) of a system of particles is identical to that of a particle of mass equal to the total mass of the system $m$ located at the CM and acted upon by a force equal to the total external force on the system” [e.g., ref. ^22^]. We next explain why this theorem anticipates the success of our 1D model.

The external force on the system can be evaluated by a judiciously chosen control volume. Consider the control volume whose top boundary is a shrink-wrap from the outside about the droplet’s liquid/gas surface and whose bottom boundary cuts through the droplet just above its wetted footprint, extending through that liquid/gas surface just above the CL, to close with the top boundary just above the CL perimeter. Such a control volume is time dependent, deforming with the droplet. At any instant during that deformation, the external contact forces acting on the drop are the CL surface tension, acting around the CL perimeter tangent to the liquid/gas surface in the direction normal to the CL tangent vector, and the bulk pressure acting on the footprint area, always normal to the planar substrate in the vertical direction.

In the absence of a body force (for small enough droplets, gravity can be neglected), for droplets deforming in a passive environment, the only external forces acting on the above control volume are contact forces. Additionally, for axisymmetric deformations, the footprint area is always circular and then, by symmetry, horizontal surface tension contributions sum to zero, leaving only a net vertical component, which combines with the pressure contribution to yield a net vertical external force. Consequently, the CM remains on the axis of symmetry and hence must execute a 1D axial motion.

For axisymmetric deformations with a changing CA and moving CL, (i) the footprint area changes, (ii) the pressure acting on that area can change, and (iii) the perimeter CL force can also change, by the changing CA. The key assumption behind the 1D reduced-order-model then is that the net effect of these external continuum forces can be described by a conservative (spring) and a non-conservative (dashpot) contribution. A main result of the paper is that frequency scanning interpreted using the 1D model allows the conservative and non-conservative pieces to be separated while the experimental contrast between CL pinned and unpinned additionally allows the non-conservative piece to be further split into a bulk and CL dissipation.

The damped harmonic oscillator of mass $m$, spring constant $k$, and damping coefficient $c$ as shown in Fig. 2b is well understood.[e.g., ref. ^23^] For a mass $m$ with displacement $Y$ connected via a spring $k$ and dashpot $c$ to a support of displacement $X$, Fig. 2b, the equation of motion can be written as

11 $$\ddot{Y}/{\omega }_{{\rm{n}}}^{2}+2\zeta \dot{Y}/{\omega }_{{\rm{n}}}+Y=2\zeta \dot{X}/{\omega }_{{\rm{n}}}+X,$$

where ${\omega }_{{\rm{n}}}=\sqrt{k/m}$ is the natural frequency, $\zeta =c/2\sqrt{km}$ is the dimensionless damping ratio and the dot denotes time derivative. Under a constant sinusoidal forcing $X=| X| \sin (\omega t)$ where $| X|$ is the forcing amplitude, $\omega$ the angular frequency of forcing (equivalent to $2\pi$ times $f$, the temporal frequency), and $t$ the time, the long-term solution is given by $Y=| Y| \sin (\omega t+\phi )$ where the amplitude $| Y|$ is constant and $\phi$ is the phase difference. We need the amplification factor $| Y/X|$ and phase difference $\phi$, given by

12a $$\left|Y/X\right|=G(\zeta ,w)\equiv \sqrt{\left\{1+{\left(2\zeta w\right)}^{2}\right\}/\left\{{\left(1-{w}^{2}\right)}^{2}+{\left(2\zeta w\right)}^{2}\right\}},$$

12b $$\tan \phi =H(\zeta ,w)\equiv -2\zeta {w}^{3}/\left\{1-\left(1-4{\zeta }^{2}\right){w}^{2}\right\},$$

where $w\equiv \omega /{\omega }_{{\rm{n}}}$. For a given $\omega$, $| Y/X|$ and $\phi$ can be measured and (12) solved for unknowns $\zeta$ and ${\omega }_{{\rm{n}}}$. The dimensionless $\zeta$ is the sought after metric for overall damping while natural frequency ${\omega }_{{\rm{n}}}$ provides a useful check against unrealistic results.

The proper interpretation of $Y$ in the droplet context is ${Y}_{{\rm{CM}}}=\beta {Y}$ but it is more convenient is to track the apex of the drop. Methods section explains this adjustment.

### ISS experiment: ISV

The inertial spreading and drop vibration (ISV) experiment planned for the ISS glove-box laboratory follows the experiment described in ref. ^19^ Briefly, a liquid drop is deposited on a solid planar substrate, which is attached to the platform of a mechanical shaker. During the experiment, a sine-wave generator sends a driving signal to the shaker, causing the platform to oscillate in the plane-normal direction. The deformation of the drop under the periodic forcing becomes more pronounced when the driving frequency is close to a resonance frequency of the drop. Sufficiently large deformations of the drop allows the contact line of the drop to unpin, giving rise to a periodic wetting-dewetting cycle. By applying the techniques described in ref. ^19^ the mobility of the contact line for the solid-liquid-gas system involved is evaluated.

The advantage of micro-gravity is in accentuating the competing effects of capillarity and inertia during these inertial spreading events. As the capillary length scale (CLS) $\sqrt{\sigma /\rho g}$ is amplified, much larger drops than possible under terrestrial gravity can be formed. By virtue of magnified drop dimensions, the capillary time-scale $\tau \equiv \sqrt{\rho V/\sigma }$ is also increased, and lower driving frequencies are needed to activate the resonant modes of interest. In other words, the spatial and temporal resolution of measurements are greatly enhanced. For the ISV experiment, drop diameters around 3 cm are used with the first axisymmetric resonance mode at ~3 Hz, which is estimated by the scaling argument that the resonance frequency of a drop scales with the inverse of ${\tau}$. In typical terrestrial experiment, drop diameter of 4 mm corresponds to a resonance frequency of about 60 Hz. Assuming the same liquid and substrate, the resonance frequency of the ISV drop is then ${f}_{{\rm{ISS}}}=f{(R/{R}_{{\rm{ISS}}})}^{3/2}$ = 3 Hz.

The basic experimental procedure contains two parts. First, a drop is oscillated under a frequency sweep to locate precisely the resonance frequencies of the [2, 0] and [4, 0] modes. Second, the drop is oscillated at each of the two resonance frequencies in turn to measure the mobility of the contact line.

### Distinguishing CL dissipation

To impose a pinned CL as a baseline for comparison, we employ a silicone pillar (P) with a 4 mm diameter. The sharp curvature at the edge of the top face prevents the CL from spreading beyond the circular edge, while the weakly hydrophilic silicone surface coupled with the volume of drop used ($V=20\ \mu {\rm{L}}$) inhibits CL retraction. There is no CL displacement observed throughout the oscillations. As a contrasting substrate, a flat silicon wafer (W) with smooth and hydrophobized surface enables CL motion under cyclic deformations of the bulk drop. See Table 4 for system designations.

**Fig. 3:**
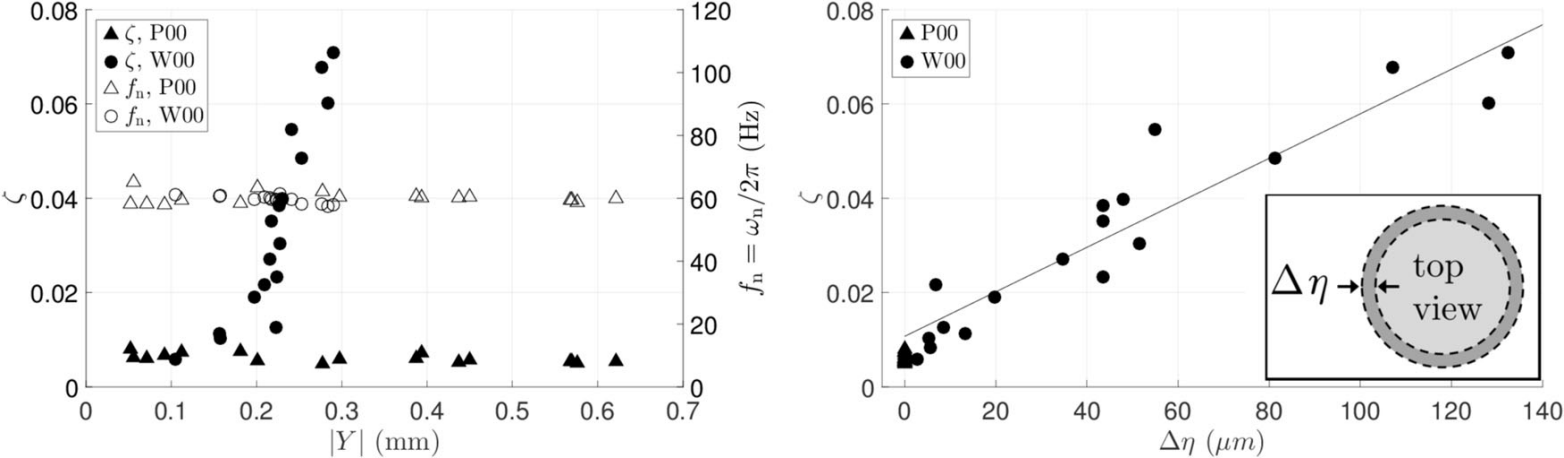
${\mathrm{P00}}$ and ${\mathrm{W00}}$ systems, 20 ${\mu}$L drops: **a** $\zeta$ and ${f}_{n}={\omega }_{{\rm{n}}}/2\pi$ against $| Y|$. **b** $\zeta$ against $\Delta \eta$. Fitting line based on W00 data. Inset: definition of $\Delta \eta$.

Figure 3a plots on the left axis the measured damping ratio $\zeta$ against the response amplitude $| Y|$. For the pillar experiments, $\zeta$ is consistently low over a ten-fold increase in driving—not surprising as water is relatively inviscid. Also, $\zeta$ is not seen to correlate strongly with driving, further validating the damping ratio as a nondimensional metric for dissipation in a drop. As for water drops on the flat wafer (${\rm {W}}00$), $\zeta$ is comparable to the pillar results when driving is weak but increases rapidly with $| Y|$ after some threshold. Over the range of our tests, $\zeta$ from the flat wafer can be up to an order of magnitude greater than that from the pillar experiments. The right axis of Fig. 3a plots the natural frequency against $| Y|$. The consistency of the calculated natural frequencies ensures that the same resonance mode is excited throughout the experiments. This is important as the damping ratio calculated here is specific to the peak at 67 Hz. Resonance at 155 Hz in Fig. 2a, for instance, would have different values of damping ratio.

Dissipation sources that could possibly contribute to the damping ratio are bulk dissipation and CL dissipation. By definition, CL dissipation must arise through CL displacement. The range of CL displacement, $\Delta \eta$, is plotted in Fig. 3b. The $\zeta$-$\Delta \eta$ relationship appears to be linear for the flat wafer experiments, showing that CL dissipation contributes strongly to the damping ratio.

### Effect of bulk viscosity

**Fig. 4:**
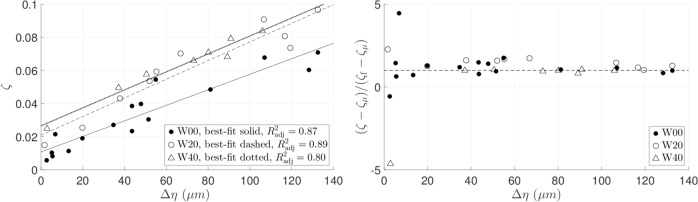
Damping ratio variation with liquid viscosity, $20\ \mu$L drops: **a** $\zeta$ against $\Delta \eta$. **b** $(\zeta -{\zeta }_{\mu })/({\zeta }_{{\rm{f}}}-{\zeta }_{\mu })$ against $\Delta \eta$. Dashed line, $\zeta ={\zeta }_{{\rm{f}}}$.

To verify that the increase in $\zeta$ with $\Delta \eta$ is directly related to CL displacement, two water-glycerol mixtures of 20 v/v% and 40 v/v% glycerol concentration are tested on the flat substrate, Fig. 4a. Again, all three lines of best-fit are approximately linear with different vertical intercepts but similar slopes. From Table 3, the 20% and 40% mixtures have, respectively, roughly twice and five times the viscosity of water. That the slopes of the three sets of data in Fig. 4a appear to be constant argues against viscous dissipation as the main contributor to the increasing trend of $\zeta$ with $\Delta \eta$. The effect of viscosity is instead manifest in the shifted vertical intercepts, where the intercept of the W20 fitting line is roughly doubled from the W00 intercept. The W40 intercept is greater yet but not quite as high as 4.83 times of W00. We attribute this shortfall to experimental uncertainty, to be discussed later.

Further post-processing using linear regression of the data in Fig. 4a as well as results from the P00 experiments is performed. First, we find equations of best-fit for each of the data sets W00, W20, and W40 in the form $\zeta =q\Delta \eta +{\zeta }_{0}$, where $q$ denotes the slope and ${\zeta }_{0}$ is the y-intercept. The mean of the three slopes, $\overline{q}$, then gives a measure of the variation of the damping ratio with CL displacement. The objective of this plot is to check the data against the fitting model ${\zeta }_{{\rm{f}}}=\overline{q}\Delta \eta +{\zeta }_{\mu }$, where ${\zeta }_{\mu }$ represents the effect of any viscous dissipation in the bulk away from the substrate and, to a lesser extent, within the thin viscous boundary layer adjacent to the substrate.

Defining ${\zeta }_{\mu ,{\rm{P00}}}$ as the mean value of $\zeta$ from the P00 data set (see Fig. 3b), we evaluate ${\zeta }_{\mu }$ according to ${\zeta }_{\mu }/{\zeta }_{\mu ,{\rm{P00}}}=\mu /{\mu }_{{\rm{P00}}}$ where $\mu$ is the dynamic viscosity. Finally, plotting $(\zeta -{\zeta }_{\mu })/({\zeta }_{{\rm{f}}}-{\zeta }_{\mu })$ against $\Delta \eta$, Fig. 4b, yields a graphical representation of the collapse of data according to this fitting. Obviously, if the fitting ${\zeta }_{{\rm{f}}}$ were exactly equal to $\zeta$, one would expect every point in Fig. 4b to fall on a horizontal line of y-value unity (dashed). Any difference between ${\zeta }_{{\rm{f}}}-{\zeta }_{\mu }$ and $\zeta -{\zeta }_{\mu }$ results in deviation from the dashed line. The errors blow up near the left-hand edge of the plot where $\Delta \eta$, and subsequently ${\zeta }_{{\rm{f}}}-{\zeta }_{\mu }$, are close to zero. However, away from there, the data largely obey the fitted behavior. The fitting parameters are given in Table 2.

**Table 2:** Fitting parameters used for Fig. 4b.

System	${\mu }_{{\rm{P00}}}$	${\zeta }_{\mu }$	$\overline{q}\times 1{0}^{3}$ ($\mu {{\rm{m}}}^{-1}$)	Max. ($\zeta -{\zeta }_{\mu }$)
${\rm{W}}00$	1	0.0065	0.47	0.064
W20	1.99	0.013	0.56	0.084
W40	4.83	0.032	0.46	0.077

See Table 4 for system definitions

### Reconciliation with mechanical work approach

The above procedure for measuring $\zeta$ is independent of the more conventional approach based on mechanical work done by $F$, c.f. Eq. (2). However, the result needs to be validated against the mechanical work approach. It turns out that the W00 system is well suited to a straightforward evaluation of mechanical work and thereby validation.

Damping $\zeta$ can be related to mechanical work using mechanical-electrical analogies.^24^ Consider the energy lost in one cycle due to CL dissipation, call it ${L}_{{\rm{CL}}}$:

13 $${\zeta }_{{\rm{CL}}}\equiv \zeta -{\zeta }_{\mu }={L}_{{\rm{CL}}}/\left\{2\pi \rho V\beta {(| Y| \omega )}^{2}\right\},$$

where ${\zeta }_{\mu }$ is the damping from non-CL contributions. See Supplementary Information for derivation. Recognizing that per-cycle losses at the CL arise from the irrecoverable mechanical work done, we put

14 $${L}_{{\rm{CL}}}=\overline{{\mathcal{P}}}\, T$$

and one finds using (Eqn. (8), left) what we will refer to as the *CL damping ratio*,

15 $${\zeta }_{{\rm{CL}}}={\mathcal{A}}[{D}_{\eta rF}]/\rho V\beta {(| Y| \omega )}^{2}.$$

For system W00, $\overline{{\mathcal{P}}}\ \ T$ can be well approximated by Eq. (10) since $\overline{\alpha }=10{1}^{\circ }\ \approx \ \pi /2$ and ${\alpha }^{* }=1{1}^{\circ }\approx 0.2\ {\rm{rad}}\ll \pi /2$ with $\sin \overline{\alpha }\approx 0.98$. This allows the unbalanced Young force Eq. (2) to be expanded in Taylor series and yields the simplification ${\mathcal{A}}[{D}_{\eta rF}]\approx \sigma \sin \overline{\alpha }\ \ {\mathcal{A}}[{D}_{{\eta }^{* }{\alpha }^{* }}]$. Hence, for W00, the contribution to damping ratio from the moving CL is proportional to the area of region ${D}_{{\eta }^{* }{\alpha }^{* }}$, the region enclosed by the periodic trajectory plotted in the plane of ${\alpha }^{* }$ against ${\eta }^{* }$.

**Fig. 5:**
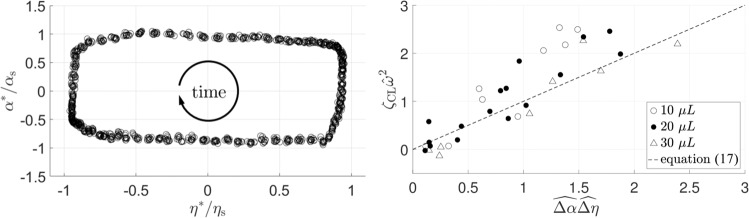
**a** Plot ${\alpha }^{* }/{\alpha }_{{\rm{s}}}$ against $\eta /{\eta }_{{\rm{s}}}$, where ${\alpha }_{{\rm{s}}}$ = 0.2 rad and ${\eta }_{{\rm{s}}}$ = 1 mm, for a typical W00 experiment, adapted from.^19^ **b** Scaling of $\zeta_{CL}$ as suggested by (17). W00 system with drop volume $V=10,20,30\ \upmu {\rm{L}}$.

A typical region ${D}_{{\eta }^{* }{\alpha }^{* }}$ for the system W00 is shown in Fig. 5a, where scaling factors ${\alpha }_{{\rm{s}}}$ and ${\eta }_{{\rm{s}}}$ are used to denote the maximum contact angle deviation and contact-line displacement, respectively, for a typical experiment. The approximately rectangular shape of this plot is not special to this particular W00 system but in fact generally expected of underdamped wetting systems with rapidly moving CLs. The enclosed area ${\mathcal{A}}[{D}_{{\eta }^{* }{\alpha }^{* }}]$ shown in Fig. 5a is approximately rectangular and thus proportional to $\Delta \alpha$ times $\Delta \eta$. The CL damping Eq. (15) then reduces to

16 $${\zeta }_{{\rm{CL}}}=R\sigma \sin \overline{\alpha }\Delta \alpha \Delta \eta /\rho V\beta | Y{| }^{2}{\omega }^{2},$$

which can be rewritten more compactly by using scaled quantities $\hat{\omega }\equiv \omega \sqrt{\rho V/\sigma }$, $\widehat{\Delta \alpha }\equiv \Delta \alpha /(| Y| /R\sin \overline{\alpha })$, and $\widehat{\Delta \eta }\equiv \Delta \eta /(\beta | Y| )$ to obtain the following dimensionless form,

17 $${\zeta }_{{\rm{CL}}}{\hat{\omega }}^{2}=\widehat{\Delta \alpha }\widehat{\Delta \eta },$$

which is plotted in Fig. 5b.

While Eq. (17) predicts a collapse of the data onto a single curve of unity slope, we see in Fig. 5b some scatter about the prediction (dashed). The scatter can be accounted for, at least in part, by the term $\beta$ Eq. (16) where it is taken to be a constant. However, $\beta$ is expected to depend weakly on the oscillation amplitude. Other uncertainties may further contribute to the scatter. Nevertheless, since Eq. (17) is a scaling argument, the collapse of the data strongly suggests that Eq. (17) captures the essential ingredients of CL dissipation. In summary, the CL dissipation according to the CL damping ratio is consistent with that based on mechanical work.

### De Gennes friction coefficient

We can now link our results back to (1) by estimating the friction coefficient, ${\mu }_{{\rm{f}}}$, in order to compare with values reported by.^13^ Above, we have introduced the per-cycle energy dissipation in terms of the de Gennes friction coefficient, Eq. (8), right. Equating Eq. ((8), right) to the per-cycle mechanical work ((8), left) yields,

18 $${\mu }_{{\rm{f}}}={\mathcal{A}}[{D}_{\eta rF}]/{\mathcal{A}}[{D}_{\eta r\dot{\eta }}]\approx (\sigma T\sin \overline{\alpha }\Delta \alpha )/(4\Delta \eta ),$$

where the approximate expression follows much as Eq. (16) follows from Eq. (15), similarly using properties of system W00.

**Fig. 6:**
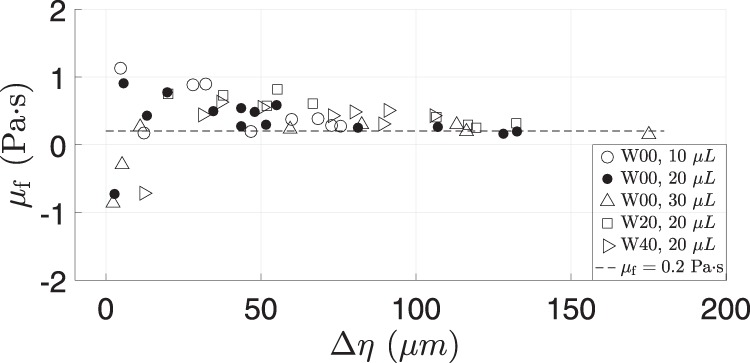
Friction coefficient, ${\mu }_{{\rm{f}}}$, according to (18).

The result on right hand side of Eq. (18) is plotted in Fig. 6. In a similar way to Fig. 4b, errors blow up for small $\Delta \eta$ but the rest of the data points tend toward a value of about 0.2 Pa s. This is in reasonable agreement with the results of ref. ^13^ who reported values of 0.1–0.2 Pa s at similar viscosity, for a selection of surfaces that encompass the equilibrium CA of the wafer (W) surface tested in this work. In comparison, for a fluorosilane-treated substrate,^19^ our calculation yields a value of ${\mu }_{{\rm{f}}}$ = 0.9 Pa s, consistent with the greater CL drag on that substrate.

Alternatively, one may relate ${\mu }_{{\rm{f}}}$ to the CL damping ratio ${\zeta }_{{\rm{CL}}}$ by putting ${L}_{{\rm{CL}}}=\overline{{\mathcal{D}}}\ \ T$, inserting into Eq. (13), and making approximation Eq. (9), to yield

19 $${\zeta }_{\rm{CL}}={\mu}_{\rm{f}}{\mathcal{A}}[{D}_{\eta \dot{\eta }}]/\rho V\beta {(\vert Y\vert \omega )}^{2}.$$

While ${\mu }_{{\rm{f}}}$ is usually taken to be a material coefficient that does not depend on the extent of contact-line motion, it is reassuring that the sensitivity of ${\zeta }_{{\rm{CL}}}$ to ${\mathcal{A}}[{D}_{\eta \dot{\eta }}]$ and hence to $\Delta \eta$ is preserved in this expression. Figure 4(d) of ref. ^19^ shows region ${D}_{\eta \dot{\eta }}$, for the system W00.

### Sources of error and advantage over direct approach

Our spatial resolution is about $10\ \upmu$m/pixel and CA measurements carry error bars of $\pm {1}^{\circ }$, as estimated by.^19^ While these uncertainties can be reduced with upgraded optics, the side-view measurements of $\eta$ yields readings for at most two azimuthal positions of the CL. Any anisotropy in the substrate, whether due to heterogeneous surface chemistry or the presence of impurities, could lead to measurements exhibiting significant departure from the averaged behaviour around the CL. However, the core of the proposed characterization has the advantage of only requiring measurement of the central height of the drop. In this way, the result is essentially isolated from uncertainties related to direct measurements near the CL. In summary, the proposed procedure based on frequency scans measures CL dissipation without needing local CA or CL measurements or any assumptions about the “equilibrium” CA.

## Discussion

Resonant-mode periodic forcing of sessile drops can excite large cyclic contact-line displacements, as center-of-mass movement along the vertical axis effectively drives sweeping CL motions along the horizontal substrate plane.

On the experimental side, the recorded oscillatory motion of the droplet peak at resonance yields the net damping ratio $\zeta$ as a reduced-order metric characterizing overall damping, Fig. 2. We introduce this straightforward measurement as a simplification, beneficial since local CA and CL measurements are avoided. Damping arising from CL dissipation ${\zeta }_{{\rm{CL}}}$ can be experimentally split off from that arising from bulk viscous effects ${\zeta }_{\mu }$, Fig. 4b, summarized by $\zeta ={\zeta }_{{\rm{CL}}}+{\zeta }_{\mu }$. For the system of water on silicon wafer (W00), we report ${\zeta }_{{\rm{CL}}}$ to be ten-fold ${\zeta }_{\mu }$, Fig. 3. An open question is the extent of the inertial-capillary regime, as defined by nondimensional parameters ranges (Table 1), for which our reduced-order-model approach remains an effective approach.

On the theoretical side, we introduce cyclic measures of the CL response. These phase-plane diagrams provide graphical representations of cyclic mechanical work done and energy dissipated at the moving CL, Eq. (8). We then relate ${\zeta }_{{\rm{CL}}}$ to the appropriate measure, Eq. (15), and test that equation, Fig. 5b. Finally, we show how the de Gennes friction coefficient can also be related to the graphical measures, Eq. (18).

## Methods

The setup is shown in Fig. 1a with relevant variables defined in Fig. 1b. Drop volumes tested include $V=10$, 20, and $30\ \mu$l, chosen to keep lengthscale below the capillary length for our Earth-bound experiments, c.f. $B{o}_{f}$, Table 1. Accordingly, rest shapes are spherical caps to good approximation and for these, for a given contact angle $\overline{\alpha }$, the footprint radius $R$ can be related to the drop volume $V$ via the formula $V=\pi {R}^{3}(2-3\cos {\overline \alpha}+{\cos }^{3}{\overline \alpha })/3 \sin^{3}{\overline \alpha}$.^25^ For $\overline{\alpha }=10{1}^{\circ }$, $R=0.72{V}^{1/3}$, for example.

Relevant properties of liquids used are given in Table 3. Designations and key characterizations of solid-liquid systems are given in Table 4.

**Table 3:** Water-glycerol mixture properties.^19^

Glycerol concentration (${\rm{v}}/{\rm{v}}\%$)	Density (${\rm{kg}}\ {{\rm{m}}}^{-3}$)	Dynamic viscosity (${\rm{m}}\,{\rm{Pa}}\,{\rm{s}}$)	Surface tension (${\rm{mN}}\ {{\rm{m}}}^{-1}$)
00	1000	1.00	71.7
20	1050	1.99	70.7
40	1100	4.83	69.1

**Table 4:** System designation combining solid ($W, \,P$) and glycerol concentration (by volume) in water ($xx \%$) where $xx$ denotes *0, 20, 40%*, (Table 3), to yield *W00*, ... *W40*, and *P00*.

Designation	Substrate	Surface treatment	Water ${\alpha }_{A}$ (${}^{\circ }$)	Water ${\alpha }_{R}$ (${}^{\circ }$)
*Wxx* (Wafer)	Silicon wafer	Trimethylsiloxy terminated PDMS	102	100
*Pxx* (Pillar)	Silicone pillar	as-cast	$>$120	$<$60

CA measured using commercial goniometer as described in ref. ^19^

### Damping ratio measurement

To measure $| Y/X|$ and $\phi$, we take high-speed measurements of $Y$ and $X$ under periodic forcing, well after any initial transient. Least-squared fits of $Y$ and $X$ to sinusoidal functions are evaluated. $| Y/X|$ and $\phi$ are then approximated as the ratio of the amplitudes and the phase difference, respectively, of the fitted sinusoids.

### $\eta$ measurement

Displacement $\eta$ is measured from the side-view profile of the drop. A RedLake HG-XL imaging system (DEL Imaging Systems, Cheshire, CT) is used for high-speed image capture with a frame rate of 5000 Hz and a typical resolution of $10\ \upmu$m/pixel.

### Replacing ${Y}_{{\rm{CM}}}$ by $Y$

**Fig. 7:**
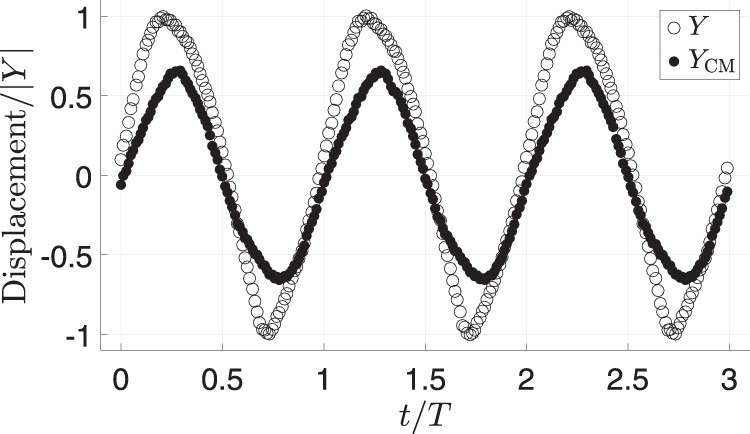
Apex displacement, ${Y}$, and ${\mathrm {CM}}$ displacement, $Y_{\mathrm{CM}}$ against time: displacement scaled by amplitude of $Y$, $| Y|$; time scaled by oscillation period, $T$.

Rather than measuring the displacement of the drop CM, we take for convenience the apex displacement as $Y$ with the assumption ${Y}_{{\rm{CM}}}=\beta Y$ where $\beta$ is a constant. Figure 7 shows ${Y}_{{\rm{CM}}}$ and $Y$ for a typical driven drop as time series over 3 periods ($T$). Here, $Y$ reasonably tracks the behavior of ${Y}_{{\rm{CM}}}$ with $\beta =0.66$ based on the amplitudes of $Y$ and ${Y}_{{\rm{CM}}}$, except for slight phase shifts near the extrema.

### Reporting summary

Further information on research design is available in the Nature Research Reporting Summary linked to this article.

## Supplementary information

Supplementary notes

Reporting Summary

## Data Availability

The datasets generated and analyzed during the current study are available from the corresponding author on reasonable request.
