# Supplementary material for: Dissipation of oscillatory contact lines using resonant mode scanning
Source: NPJ Microgravity. 2020 Jan 21;6:3. doi: 10.1038/s41526-019-0093-0 (PMC6972899; doi:10.1038/s41526-019-0093-0)
Supplement: Supplementary file 1 — Supplementary notes [file 41526_2019_93_MOESM1_ESM.pdf]

Supplementary Notes

To derive equation 13 of the main document, we begin with an argument involving the center-of-mass displacement $Y_{\rm CM} = |Y_{\rm CM}| \sin(\omega t + \phi)$. Following that, a result relating the damping ratio based on center-of-mass displacement to that based on drop apex displacement is derived. Finally, we combine the results to obtain the desired equation.

The CM of an axially symmetric deformation, $Y_{\rm CM}$, remains on the symmetry axis and hence the droplet kinetic energy of CM motion can be written in terms of the CM velocity (dot denotes time derivative) as,

$$\mathcal{K}(t) = \rho V \ \dot{Y}_{\rm CM}^2/2, \tag{1}$$

where $\rho V = m$ for our drops of constant density ρ and volume V.

Damping ratio and quality factor

The damping ratio based on the drop center of mass (CM), $\zeta_{\rm CM}$, can be related to dimensional measures of energy dissipation using notion of the "quality-factor" Q, arising from classical mechanical-electrical analogies (1). The relationship is $2Q\zeta_{\rm CM} = 1$, where $Q/2\pi$ is the per-cycle maximum energy stored relative to the energy lost in an oscillator.

Noting that $\dot{Y}_{\rm CM} = |Y_{\rm CM}|\omega \sin(\omega t + \phi) \leq |Y_{\rm CM}|\omega$, which is attained during the cycle, the maximum droplet stored energy can be expressed in terms of the kinetic energy (1) as $\rho V(|Y_{\rm CM}|\omega)^2/2$. Using the definition of Q and adopting the notation L for energy lost in one cycle, we start with,

$$\zeta_{\rm CM} = L/\left\{2\pi\rho V(|Y_{\rm CM}|\omega)^2\right\} = (L_{\rm CL} + L_\mu)/\left\{2\pi\rho V(|Y_{\rm CM}|\omega)^2\right\}, \tag{2}$$

where L_{CL} denotes energy lost in one cycle to CL dissipation and L_μ denotes energy lost in one cycle to viscous dissipation elsewhere.

The damping ratio can be split into contributions from CL dissipation $\zeta_{\mathrm{CM,CL}} = L_{\mathrm{CL}}/2\pi\rho V(|Y_{\mathrm{CM}}|\omega)^2$ and from viscous dissipation elsewhere $\zeta_{\mathrm{CM},\mu} = L_\mu/2\pi\rho V(|Y_{\mathrm{CM}}|\omega)^2$. It follows that $\zeta_{\mathrm{CM}} = \zeta_{\mathrm{CM,CL}} + \zeta_{\mathrm{CM},\mu}$, or equivalently,

$$\zeta_{\mathrm{CM}} - \zeta_{\mathrm{CM},\mu} = L_{\mathrm{CL}}\big/\left\{2\pi\rho V(|Y_{\mathrm{CM}}|\omega)^2\right\}\,, \tag{3}$$

which is the center of mass version of relationship (13).

Relating ζ_{CM} to ζ

From classic vibrations theory (e.g. 2), for $\zeta \ll 1$, the amplification factor $|Y_{\mathrm{CM}}/X|$ at the resonant frequency is given by

$$|Y_{\mathrm{CM}}/X| \approx (1/2\zeta_{\mathrm{CM}})\left(1 + 5\zeta^2/2\right) \approx 1/2\zeta_{\mathrm{CM}}, \tag{4}$$

to first order. Similarly, $|Y/X| \approx 1/2\zeta$.

Using the approximation $Y_{\mathrm{CM}} = \beta Y$ as proposed in the main document,

$$\zeta_{\mathrm{CM}} \approx 1/(2|Y_{\mathrm{CM}}/X|) \approx 1/(2\beta|Y/X|) \approx 1/\{2\beta(1/2\zeta)\} \approx \zeta/\beta. \tag{5}$$

For $\zeta_{\mathrm{CM},\mu}$, we assume an expression of the same form but with β_μ instead of β at first to acknowledge that the resonant frequency associated with the overall dissipation might be different from that associated with viscous damping away from the CL. As it turns out, for $\zeta \ll 1$, the resonant frequency is given approximately by $\omega_{\mathrm{res}}/\omega_{\mathrm{n}} = 1 - \zeta^2$ and similarly, $\omega_{\mathrm{res},\mu}/\omega_{\mathrm{n}} = 1 - \zeta_\mu^2$, where we use $\omega_{\mathrm{res},\mu}$ to denote the resonant frequency associated with viscous damping away from the CL. Since $\zeta_\mu < \zeta < 0.1$, $\omega_{\mathrm{res},\mu} \approx \omega_{\mathrm{res}}$, and it follows that $\beta_\mu \approx \beta$.

In summary,

$$\zeta_{\mathrm{CM},\mu} \approx \zeta_\mu/\beta. \tag{6}$$

Final derivation of (13)

Substituting (5) and (6) into (3), we have finally

$$\zeta_{CL} = L_{\mathrm{CL}}\big/\left(2\pi\rho V\beta|Y|^2\omega^2\right)\ , \tag{7}$$

which was presented in the main document as (13).

References

[1] Siebert, W. M. *Circuits, Signals, and Systems* (The MIT Press, 1985).

[2] Den Hartog, J. P. *Mechanical Vibrations* (Dover Publications, New York, 1985).
